# Protein Domain Analysis of Genomic Sequence Data Reveals Regulation of LRR Related Domains in Plant Transpiration in *Ficus*

**DOI:** 10.1371/journal.pone.0108719

**Published:** 2014-09-30

**Authors:** Tiange Lang, Kangquan Yin, Jinyu Liu, Kunfang Cao, Charles H. Cannon, Fang K. Du

**Affiliations:** 1 Key Laboratory of Tropical Forest Ecology, Xishuangbanna Tropical Botanical Garden, Chinese Academy of Sciences, Menglun, Mengla, Yunnan Province, China; 2 College of Forestry, Beijing Forestry University, Beijing, China; 3 School of Life Science, Tsinghua University, Beijing, China; 4 State Key Laboratory for Conservation and Utilization of Subtropical Agro-Bioresources, and College of Forestry, Guangxi University, Nanning, Guangxi, China; 5 Department of Biological Sciences, Texas Tech University, Lubbock, Texas, United States of America; Shenzhen Institutes of Advanced Technology, China

## Abstract

Predicting protein domains is essential for understanding a protein’s function at the molecular level. However, up till now, there has been no direct and straightforward method for predicting protein domains in species without a reference genome sequence. In this study, we developed a functionality with a set of programs that can predict protein domains directly from genomic sequence data without a reference genome. Using whole genome sequence data, the programming functionality mainly comprised DNA assembly in combination with next-generation sequencing (NGS) assembly methods and traditional methods, peptide prediction and protein domain prediction. The proposed new functionality avoids problems associated with *de novo* assembly due to micro reads and small single repeats. Furthermore, we applied our functionality for the prediction of leucine rich repeat (LRR) domains in four species of *Ficus* with no reference genome, based on NGS genomic data. We found that the LRRNT_2 and LRR_8 domains are related to plant transpiration efficiency, as indicated by the stomata index, in the four species of *Ficus*. The programming functionality established in this study provides new insights for protein domain prediction, which is particularly timely in the current age of NGS data expansion.

## Introduction

With the advent of next-generation sequencing (NGS) technology, a massive amount of DNA data is currently being produced in both model and non-model species. However, there are many problems associated with *de novo* assembly, i.e., when there is no reference genome on which to map reads, especially when the genome structure is complex with large parts of repetitive elements, as it is often the case in plant species [1]. In such cases, the DNA reads can only be assembled to scaffold or contig level [2]. Thus, methods based on an analysis of the fragments are needed.

A protein domain is a conserved part of a protein sequence which has a specific structure and function. The typical length of a protein domain is from about 25 to 500 amino acids. For some protein domain analysis, the whole protein sequence is not required [3]. Hence, some of the problems associated with full-length assembly without a reference genome can be avoided by protein domain analysis.

In the present study, fig trees belonging to the *Ficus* genus of the Moraceae family were examined to verify the above hypothesis. The *Ficus* genus has been found to have great diversity in tropical and subtropical areas, which is linked to geographical evolution within the genus [4], [5]. *Ficus altissima* Blume, *Ficus tinctoria* G. Forst, *Ficus langkokensis* Drake and *Ficus fistulosa* Reinw. ex Blume usually have overlapping distributions. However, their ecological niches are different due to their physiology. *F. altissima* and *F. tinctoria* are semi-epiphytic and their leaves are coriaceous. As a result, they can tolerate environments with drought episodes [6]. In contrast, *F. langkokensis* and *F. fistulosa* grow in relatively humid habitats, such as waterside rocks, and their leaves are thin coriaceous [7]. The ecological differences in the growing areas of these different *Ficus* species might thus exert different types of drought stress pressures, leading to different responses in stomatal development and morphology [8]. Hence, it would be valuable to develop a model that predicts the peptide domains of proteins for genes potentially involved in responses to drought stress, using genomic data.

**Figure 1:**
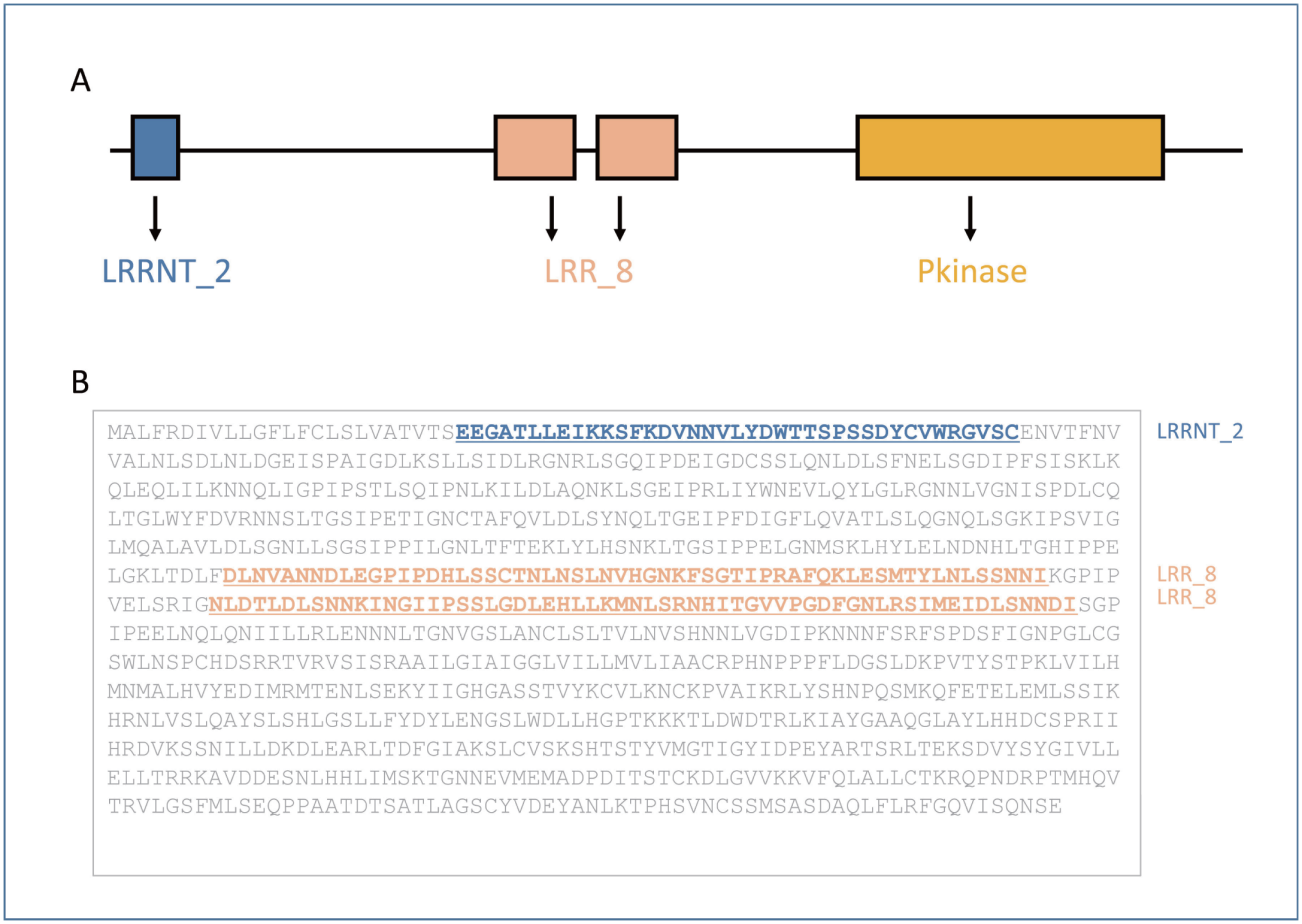
Protein domain structure of the protein encoded by the *ERECTA* gene in *Arabidopsis thaliana.* A. From the N- to C-terminal, the protein is composed of one LRRNT_2 domain, two LRR_8 domains and one Pkinase domain. B. Amino acids of the protein. The LRRNT_2 domain and two LRR_8 domains are underlined. Leucine repeats can be found in the latter domains.

One of the strategies used by plants to respond to drought stress events is plant transpiration efficiency. In the model plant *Arabidopsis*, plant transpiration efficiency is a quantitative trait, which has been shown to be controlled by several genes based on quantitative trait loci (QTLs) mapping studies [9]. To date, only a few contributing genes have been identified, one of which is the *ERECTA* gene, which explains 21–46% of the total phenotypic variation in Δ(leaf carbon isotopic discrimination) [9]. In *Arabidopsis*, ERECTA is one of the best studied receptor like kinases (RLKs) with leucine rich repeat (LRR) domains, which not only participates in plant transpiration efficiency but also regulates aerial architecture, stomatal patterning and confers resistance to the pathogenic bacteria *Ralstonia solanacearum*, the necrotrophic fungi *Plectosphaerella cucumerina* and *Pythium irregulare* [10], [11]. Structurally, the protein encoded by the *ERECTA* gene in *Arabidopsis* has one LRRNT_2 protein domain at the N-terminal, two LRR_8 protein domains in the middle part, and one Pkinase domain at the C-terminal (Fig. 1A). The LRR_8 domains form the hydrophobic core of the proteins, and they are frequently involved in the formation of protein-protein interactions [11], [12]. The LRRNT_2 domain of the protein encoded by *ERECTA* in *Arabidopsis* has LRRs flanked by cysteine rich sequences (Fig. 1B).

In contrast to model species, the molecular mechanism of plant transpiration efficiency still remains unclear in many plant and tree species, especially those without reference genomes. Improving functional annotation of assembled data obtained from NGS technology may provide new insights into genes potentially involved in this important trait. In this study, our first objective was to develop a method for obtaining high quality contigs from low coverage NGS data. Secondly, we attempted to predict protein domains from contigs obtained via the above method. Finally, we utilized our programming functionality to predict LRR domains homologous to those from the *Arabidopsis ERECTA* gene in four *Ficus* species that respond differently to drought environments and examined the relationship between LRR domain numbers and plant transpiration efficiency.

## Materials and Methods

### DNA extraction and genome sequence

Leaf material of four species, *F. altissima*, *F. tinctoria*, *F. langkokensis* and *F. fistulosa*, was collected from the Xishuangbanna Tropical Botanical Garden, Yunnan Province, P. R. China (101°25′E, 21°41′N) in April 2013 and stored in a paper bag with silica gel until DNA extraction. The four species had been transplanted in 1990 from the natural Xishuangbanna Tropical Forest, Yunna Province, P. R. China (101°57′E, 21°48′N). Genomic DNA of each individual was extracted from dried leaves using the DNeasy Plant Kit (Qiagen). DNA quality was checked on 2% agarose gels stained with ethidium bromide using a UV-Vis spectrometer (Bio-Rad Molecular Imager ChemiDoc XRS+ Imaging System) coupled with a Qubit fluorometer (ds DNA BR, Invitrogen). 40 ug RNA-free genomic DNA were used for the library construction. Library preparation (400-bp and 150-bp paired-end reads) and sequencing on an Illumina HiSeq2000 were performed by the Beijing Genomics Institute.

### Quality control methods

The raw data from the Illumina Hiseq2000 were trimmed with two programs for performing quality control written in the Practical Extraction and Report Language (PERL). The first program was used to remove nucleotides with a Phred score lower than 20 (Script S1). The second program was used to delete fastq reads with length less than 20 base pairs as well as “orphanage” reads (single reads not in a pair) created by the first program (Script S2).

### Sequence assembly

To generate a better genome assembly, we used a combination of four popular assembly software packages: ABySS, SOAPdenovo, Velvet and Phrap. ABySS, SOAPdenovo and Velvet were used to align the trimmed Illumina fastq reads to obtain contigs. These contigs were then aligned again with Phrap to improve the alignment.

First of all, ABySS (http://www.bcgsc.ca/platform/bioinfo/software/abyss) which allows *de novo*, parallel, paired-end sequence assembly for short sequence reads was used to construct alignments [13]–[15] on our *Ficus* genome data. We employed 25 as the k-mer length and 10 as the minimum number of pairs needed for two joined contigs.

Secondly, SOAPdenovo (http://soap.genomics.org.cn/soapdenovo.html) [16], which is particularly designed to assemble Illumina GA short reads, was used for building the contigs. The detailed parameter set was as follows: k-mer length 25; average insert size 250; cutoff value of pair number for a reliable connection between two contigs of pre-scaffolds 3; and minimum alignment length between a read and contig required for a reliable read location 32.

Thirdly, Velvet (http://www.ebi.ac.uk/~zerbino/velvet/) [17], which is a sequence assembler for very short sequence reads, was also applied for the sequence alignment. We set the k-mer length as 25 and the average insert size as 250.

Finally, Phrap (http://www.phrap.org/) [18], which is a program for assembling shotgun DNA sequence data was further applied on the sequence to increase the maximum length and remove redundancy. We analyzed the results of ABySS, SOAPdenovo and Velvet by Phrap (for parameters see Table S1 and some connection Script S3).

### Gene structure identification

GENSCAN (http://genes.mit.edu/GENSCANinfo.html) was used to identify complete gene structures in genomic DNA. It is a GHMM-based program that can be used to predict the location of genes and their exon-intron boundaries in genomic sequences are from a variety of organisms. The “Arabidopsis.smat” file was downloaded and used as parameter file for the *Ficus* genome data [19].

### Protein domain prediction

HMMER 3.0 (http://hmmer.janelia.org/) was used for searching sequence databases for homologs of protein sequences and making protein sequence alignments [20]. It employs methods using probabilistic models called profile hidden Markov models (profile HMMs). We used hmmscan to predict protein domains in the gene *ERECTA*, which were then predicted in *Ficus* and *Populus* by hmmsearch.

### Experimental analysis

#### Stomata index evaluation

The study was conducted in the Xishuangbanna Tropical Botanical Garden in Yunnan Province, P. R. China (101°25′E, 21°41′N) in August 2013. Four to six trees of each species showing good growth performance were sampled. We collected three mature and well-exposed leaves from each tree. To obtain a better view of the stomata, we removed the main vein of leaves and then boiled them in hot alkaline buffer to remove the mesophyll. Treated leaves were examined under a light microscope (DM2500, Leica, Germany). The numbers of stomata and epidermal cells were counted using ImageJ (National Institutes of Health, Bethesda, MD, USA, http://rsb.info.nih.gov/ij/index.html). We used the formula (100×stomatal density)/(stomatal density + epidermal cell density) to calculate the stomata index as in Hara *et al*., (2009) [21]. We repeated the experiments six times.

## Results and Discussion

### Genomic contigs assembled from Illumina genomic sequence data

**Figure 2:**
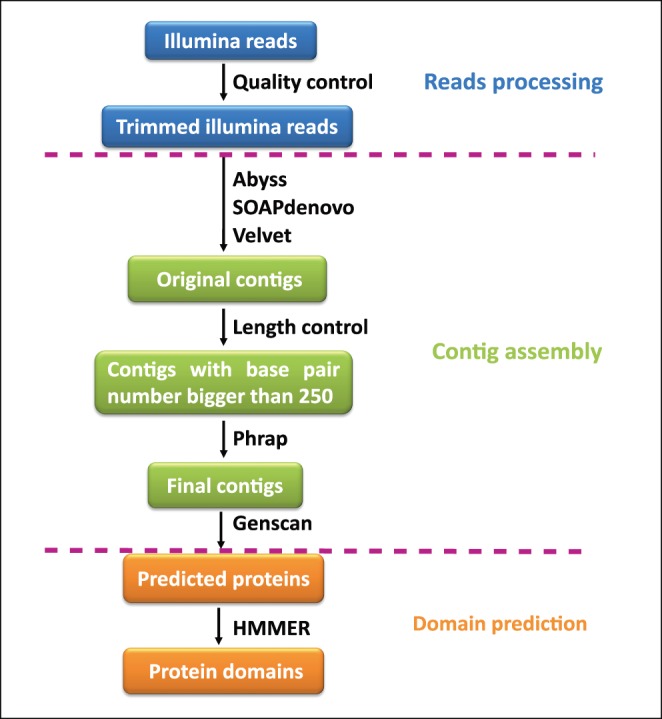
The proposed programming functionality for predicting protein domains directly from genomic sequence data without a reference genome. The Illumina reads were first trimmed with quality control methods. Then, assembly software ABySS, SOAPdenovo and Velvet were used separately to obtain original contigs. Next, length control methods were used to select contigs larger than 250 base pairs. Afterwards, the assembly software Phrap was used to obtain final contigs and Genscan was used to predict peptides from these contigs. Finally, Hmmsearch was used to predict protein domains.

The Illumina Hiseq2000 genomic sequence data in fastq format for each of *F. altissima, F. tinctoria, F. langkokensis* and *F. fistulosa* was about 15 Gigabytes, and thus the coverage was about 15x [22]. The quality control methods improved the quality of the Illumina genomic sequence data for all four species, where the number of reads was about 135 million for each species. For the gene and protein domain prediction, it was most appropriate to use the contigs that had a base pair number >250 as the input for Phrap as the typical number of nucleotides in a DNA fragment encoding a protein domain of the LRR family is about 250 (Fig. 2). The maximum lengths of contigs predicted by Phrap in *F. altissima, F. tinctoria, F. langkokensis* and *F. fistulosa* were 6914 base pairs, 10755 base pairs, 6665 base pairs and 9203 base pairs, respectively. These numbers showed that the genomic contigs finally assembled could be used for the gene prediction (Table 1).

**Table 1:** Results from the assembly software.

Species	#fastqreads	Coverage	Software	#contig_250	max_len(bp)	#pep	max_len(aa)	#LRRNT_2	#LRR_8
FA	2,185,253,886	4.86	Abyss	26,816	1,968	10,846	606	5	72
			SOAP	26,898	1,906	10,735	578	11	71
			Velvet	123,763	6,407	23,086	702	22	120
			Phrap	114,596	6,914	21,901	880	19	132
FT	2,197,543,362	4.88	Abyss	54,144	2,739	8,595	436	3	40
			SOAP	59,831	2,524	8,419	306	4	33
			Velvet	170,753	9,251	15,319	418	6	59
			Phrap	154,710	10,755	14,807	467	9	62
FL	1,993,136,266	4.43	Abyss	7,679	2,002	2,426	506	1	24
			SOAP	8,321	3,430	2,611	506	3	23
			Velvet	86,717	6,718	6,479	534	3	32
			Phrap	84,287	6,665	6,822	550	3	45
FF	869,615,244	1.93	Abyss	7,087	5,558	2,669	772	2	14
			SOAP	7,049	7,064	2,609	772	0	12
			Velvet	12,129	7,511	3,092	772	0	17
			Phrap	13,972	9,203	3,827	1,536	2	19

FA, FT, FL and FF stands for *Ficus altissima, Ficus tinctoria, Ficus langkokensis* and *Ficus fistulosa*, respectively.

#fastq reads: number of fastq reads from Illumina Hiseq2000.

#contig_250: number of predicted contigs longer than 250 base pairs.

max_len (bp): number of base pairs (bp) of the contigs predicted with maximum length.

#pep: number of peptides predicted.

max_len (aa): number of amino acids (aa) of the peptides predicted with maximum length.

#LRRNT_2: number of LRRNT_2 domains predicted.

#LRR_8: number of LRR_8 domains predicted.

### Peptides and protein domains predicted from genomic contig data

Genscan was used to predict peptide sequences from the genomic contigs assembled by Phrap for *F. altissima, F. tinctoria, F. langkokensis and F. fistulosa* (Fig. 2). The maximum lengths of peptides predicted by Genscan for the four species (880, 467, 550 and 1576 amino acids, respectively) were employed since longer peptides enable better protein domain prediction. These numbers indicated that the predicted peptides could be used to predict protein domains (Table 1).

HMMER was used to predict protein domains from the peptides predicted from Genscan for the four species (Fig. 2). The numbers of LRRNT_2 domains predicted from the contigs assembled by Phrap were 19, 9, 3, and 2 in *F. altissima, F. tinctoria, F. langkokensis and F. fistulosa*, respectively, whereas the numbers of LRR_8 domains were 132, 62, 45, and 19, respectively (Table 1).

### Phrap can improve assembly of contigs and remove identical segments of genomic sequences

**Figure 3:**
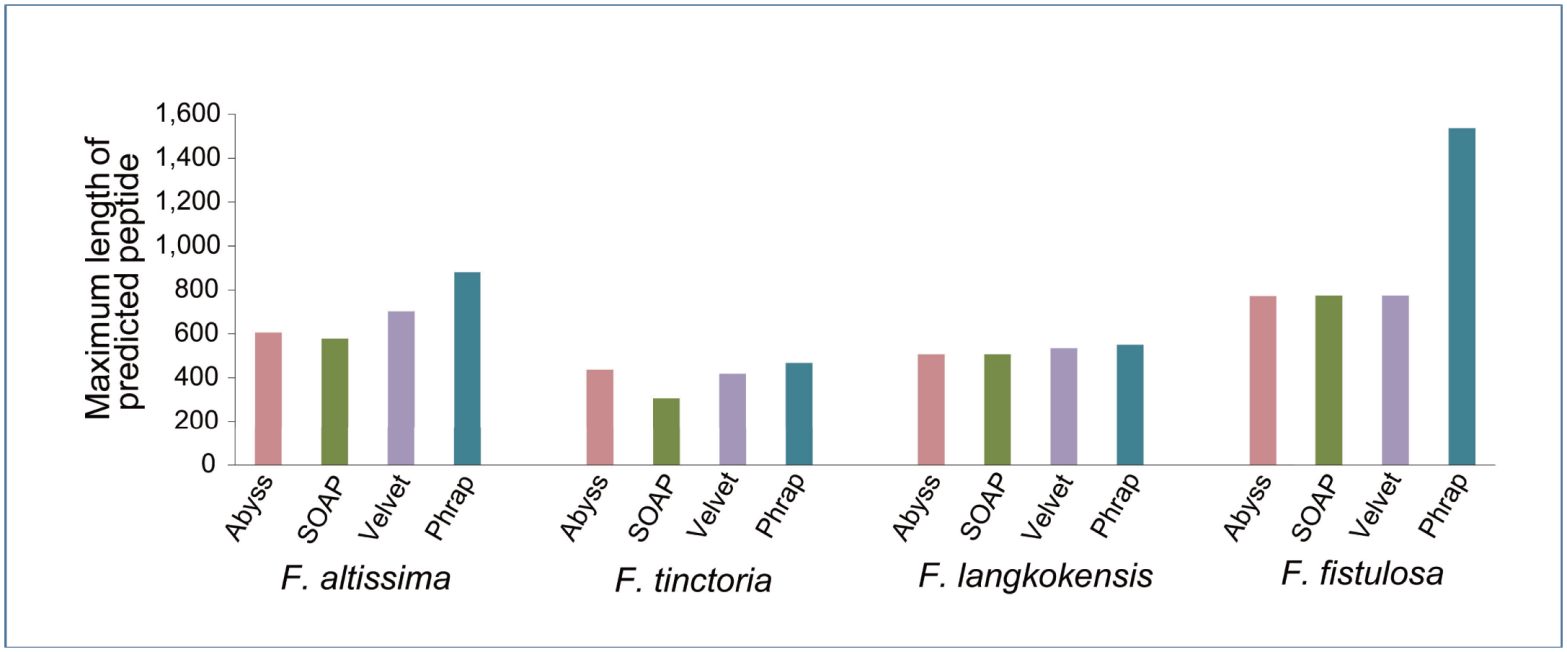
Maximum length (number of amino acids) of peptides predicted by the programming functionality. The Illumina reads for *F. altissima (FA), F. tinctoria (FT), F. langkokensis (FL)* and *F. fistulosa (FF)* were assembled by ABySS, SOAPdenovo and Velvet. Phrap was used to assemble the contigs from ABySS, SOAPdenovo and Velvet, and then Genscan was used to predict peptides from these contigs. The maximum length of the peptides could be increased by Phrap in *FA*, *FT*, *FL* and *FF*.

Phrap cannot directly work on Illumina fastq reads. However, it can increase the maximum length of the contigs assembled through ABySS, SOAPdenovo and Velvet, which can directly work on Illumina fastq reads (Table 1). Thus, the maximum length of the peptides predicted by Genscan may also be increased (Fig. 3).

Assembly through Phrap can remove redundancy of the contigs, including the identical genomic sequence segments predicted by ABySS, SOAPdenovo and Velvet. The percentage of redundancy removed from *F. altissima*, *F. tinctoria*, *F. langkokensis* and *F. fistulosa* was 36.51, 45.91, 18.64 and 46.91 respectively (Table 2). The Phrap software can be used to combine identical DNA fragments into one sequence, thus avoid the effect of gene expression difference produced by NGS methods. Programs like CAP3 and TIGR Assembler may also offer similar functions. However, it is important to choose correct parameters for different species. When applying on the contigs which are assembled by NGS assembly methods, we found that Phrap has more suitable parameters to be adjusted comparing to other programs by testing different combinations of parameters values. The raw data sequences used here were submitted to NCBI under accession number SRP041276.

**Table 2:** Redundancy removed by Phrap.

Species	#contig_250from Abyss,SOAP andVelvet	#basepairs	#contig_250not usedby Phrap	#basepairs	#contig_250used byPhrap	#basepairs	#contig_250after Phrap	#basepairs	Percent ofredundancy removedby Phrap
FA	177477	79652086	86943	37241030	90534	42411056	27653	13333742	36.51
FT	284698	124099037	95706	40299597	188992	83799440	59004	26821922	45.91
FL	102717	40680387	75107	29210626	27610	11469761	9180	3886983	18.64
FF	26265	9447858	7416	2344792	18849	7103066	6556	2670964	46.91

FA, FT, FL and FF stands for *Ficus altissima, Ficus tinctoria, Ficus langkokensis* and *Ficus fistulosa*, respectively.

#contig_250: number of contigs longer than 250 base pairs.

#base pairs: number of base pairs.

### The numbers of LRRNT_2 and LRR_8 domains in *Ficus* correlate with stomata index values

**Figure 4:**
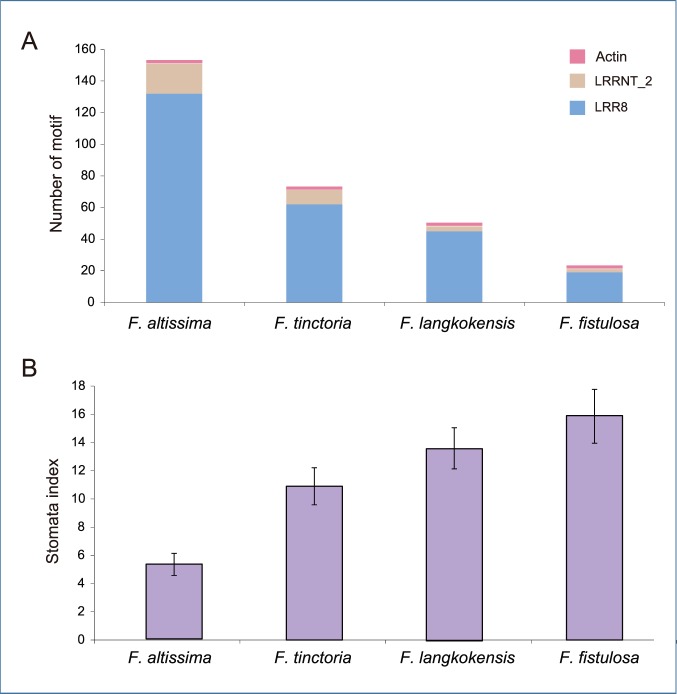
Number of LRRNT_2, LRR_8 and actin domains predicted in *F. altissima (FA), F. tinctoria (FT), F. langkokensis (FL)* and *F. fistulosa (FF)* (A); and stomata index in *FA*, *FT*, *FL* and *FF* (B). As the number of LRRNT_2 and LRR_8 domains decreased for *FA*, *FT*, *FL* and *FF*, the stomata index increased.

To test whether the LRRNT_2 domains and LRR_8 domains are related to transpiration efficiency, we used our programming functionality to predict their numbers in the four species of *Ficus*. The mean values of the stomata index for *F. altissima, F. tinctoria, F. langkokensis and F. fistulosa* were 5.3, 10.9, 13.6 and 15.9, respectively (Table 3). As the stomata index values increased in these species the numbers of LRRNT_2 and LRR_8 domains decreased accordingly (Fig. 4). To eliminate the contingency in protein domain selection, we used the actin domain from actin1 protein in *Arabidopsis thaliana* (NCBI accession number NP_850284.1) for control analysis. Actin is a house-keeping protein expressed in every plant cell as a component of the cytoskeleton [23], and thus provides a good control. Among all the peptides predicted for the four *Ficus* species, one actin domain was found to be longer than 100 amino acids and another was shorter than 50 amino acids (Fig. 4). These results suggest that the transpiration efficiency could be related to the LRRNT_2 and LRR_8 domains in *Ficus*.

**Table 3:** Physiological, anatomical and stomata response data in *Ficus*.

Species		#stomata	#epidermalcells	Stomataldensity	Epidermalcell density	Stomatalindex
FA	M	12.91667	231.1944	326.5458	5844.819	5.301273
	SD	2.061553	20.15769	52.11805	509.606	0.79305
	SE	0.343592	3.359615	8.686342	84.93433	0.132175
FT	M	20.84848	169.0303	527.0699	4273.25	10.90198
	SD	4.016538	13.41754	101.542	339.2083	1.331365
	SE	0.699189	2.335693	17.67619	59.04859	0.231761
FL	M	15.66667	99.47619	396.0685	2514.854	13.61349
	SD	1.932184	7.35268	48.84747	185.8829	1.467321
	SE	0.421637	1.604486	10.65939	40.56297	0.320196
FF	M	19.125	99.2	483.4985	2507.872	15.90947
	SD	3.879433	10.0584	98.07582	254.2861	1.932021
	SE	0.969858	2.597068	24.51895	65.65639	0.498846

FA, FT, FL and FF stands for *Ficus altissima, Ficus tinctoria, Ficus langkokensis* and *Ficus fistulosa*, respectively.

M, SD, and SE: mean, standard deviation and standard error, respectively.

#stomata: number of stomata.

#epideman cells: number of epidermal cells.

The *ERECTA* gene has not only a positive regulatory role on respiration in drought conditions but also benefits plants in the absence of water shortage [9]. Therefore, the protein domains in the *ERECTA* gene might show a cumulative positive evolution. The LRR_8 domain has more LRRs than the LRRNT_2 domain, and thus may have a more important role in protein-protein interactions (Fig. 1). Hence, this could explain why the number of LRR_8 domains was more than that of LRRNT_2 domains (Fig. 4).

## Conclusion

The programming functionality in this study was proved to be a useful tool in biological studies by showing that the LRRNT_2 and LRR_8 domains were potentially related to plant transpiration efficiency, as we can see from the stomata index in *F. altissima*, *F. tinctoria*, *F. langkokensis*, and *F. fistulosa*. The main benefit of the functionality is that it overcomes many of the complex problems associated with *de novo* assembly. However, with the increasing read lengths produced by NGS and improvements in third-generation sequencing, such problems may also be solved with the rapid developments of *de novo* assembly methods. The main limitation of the functionality is GENSCAN prediction step, which requires a suitable model. In addition, it is hard for some species to choose a perfect model to predict the gene structure. Confronting with this situation, researchers normally prefer to pick a widely used model which turns out to have more or less shortage. Nevertheless, methods of whole genome protein domain analysis will still help researchers to better understand some mechanisms of biological function from the perspective of genetic sequence, if combined with a large amount of NGS data.

## Supporting Information

Table S1 **Table for Phrap parameters.**
(DOCX)

Script S1 **Perl program used to remove the nucleotides which have Phred score lower than a specific value.**
(DOCX)

Script S2 **Perl program used to delete the fastq reads which have length less than a specific value as well as to erase the “orphange” reads (single reads without pair).**
(DOCX)

Script S3 **Perl programs used for dealing with the assembly files which were created by Phrap as well as for making statistic analysis.**
(DOCX)
