# Supplementary material for: Protein Domain Analysis of Genomic Sequence Data Reveals Regulation of LRR Related Domains in Plant Transpiration in Ficus
Source: PLoS One. 2014 Sep 30;9(9):e108719. doi: 10.1371/journal.pone.0108719 (PMC4182558; doi:10.1371/journal.pone.0108719)
Supplement: Table S1 — Table for Phrap parameters. (DOCX) [file pone.0108719.s001.docx]

**Table S1**

| Quality value to be used for each base | Trim start value | Value of relaxed stringency to varying degrees during the final contig merge pass | Value of controls treatment of inconsistent reads in merge | Value of maximum permitted size of an unmatched region in merging contigs during first merging pass | Value of controls stringency of match required for joins | Value of minimum segment size | Value of spacing between modes | Value of cutoff for flagging “low quality” regions in contig sequence and “high quality” discrepancies between read and contig |
| --- | --- | --- | --- | --- | --- | --- | --- | --- |
| 15 | 0 | 0 | 1 | 30 | 0.7 | 8 | 4 | 20 |
| Value of maximum subclone size for forward-reverse read pair consistency check | Value of minimum score for identifying degenerate sequence at beginning or end of read | Value of penalty used for identifying degenerate sequence at beginning or end of read | Quality value used to define the “high-quality” part of a read | Value of minimum size of confirming segment | Value of amount by which confirming segments are trimmed at edges | Value of penalty used in aligning against “confirming” reads | Value of minimum alignment score for a read to be allowed to “ confirm” part of another read | Value of size of indexing words used in finding word matches between sequences |
| 5000 | 20 | -2 | 13 | 8 | 1 | -5 | 30 | 30 |

Supplementary Table 1. Phrap parameters. This software was used on the output sequences of next-generation sequencing assembly softwares ABySS, SOAPdenovo, and Velvet. The first and third rows list parameter meanings. The second and fourth rows list parameter values corresponding to each parameter meaning in each cell of the first and third rows, respectively.
