# Supplementary material for: Protein Domain Analysis of Genomic Sequence Data Reveals Regulation of LRR Related Domains in Plant Transpiration in Ficus
Source: PLoS One. 2014 Sep 30;9(9):e108719. doi: 10.1371/journal.pone.0108719 (PMC4182558; doi:10.1371/journal.pone.0108719)
Supplement: Script S1 — Perl program used to remove the nucleotides which have Phred score lower than a specific value. (DOCX) [file pone.0108719.s002.docx]

**Script S1**

::::::::::::::

fastqTrim_Quality.pl: This program was used to remove the nucleotides which have Phred score lower than a specific value.

::::::::::::::

#!/usr/bin/env perl

use strict;

use warnings;

use Getopt::Long;

use File::Spec;

my $usage = "

$0 input_files [-p|probcutoff 0.05] [-h|phredcutoff 13] [-b|bwa] [-d|directory path] [-sanger -solexa -illumina] [-454]\n

-p|probcutoff probability value (between 0 and 1) at which base-calling error is considered too high (default; p = 0.05) *or*

-h|phredcutoff Phred score (between 0 and 40) at which base-calling error is considered too high

-b|bwa use BWA trimming algorithm

-d|directory path to directory where output files are saved

-sanger Sanger format (bypasses automatic format detection)

-solexa Solexa format (bypasses automatic format detection)

-illumina Illumina format (bypasses automatic format detection)

-454 set flag if trimming Roche 454 data (experimental feature)

\n";

if( !$ARGV[0] ){ die "$usage"; }

my $prob_cutoff;

my $phrd_cutoff;

my $ascii_cutoff;

my $automatic_detection_lines = 10000;

my $sanger;

my $solexa;

my $illumina;

my $format;

my $user_defined;

my $bwa;

my $directory;

my $roche;

my $poor_quality_char = "B";

GetOptions(

"p|probcutoff=f" => \$prob_cutoff,

"h|phredcutoff=f" => \$phrd_cutoff,

"b|bwa" => \$bwa,

"d|directory=s" => \$directory,

"sanger" => \$sanger,

"solexa" => \$solexa,

"illumina" => \$illumina,

"454" => \$roche

);

if( ($sanger && $solexa) || ($sanger && $illumina) || ($solexa && $illumina) ){

die "error: please select only one of -sanger, -solexa or -illumina\n";

}

if( $sanger || $solexa || $illumina ){

$user_defined = 1;

}

if( $sanger ){

$format = "sanger";

}elsif( $solexa ){

$format = "solexa";

}elsif( $illumina ){

$format = "illumina";

}

if( $roche ){

$format = "sanger";

}

my @files = @ARGV;

if( !$files[0] ){ die "$usage"; }

if( !defined( $prob_cutoff ) && !defined( $phrd_cutoff ) ){

$prob_cutoff = 0.05;

print STDOUT "Info: Using default quality cutoff of P = $prob_cutoff (change with -p or -h flag)\n";

}elsif( defined( $prob_cutoff ) && defined( $phrd_cutoff ) ){

die "Error: Please enter either a probability or a Phred quality cutoff value, not both";

}elsif( defined( $prob_cutoff ) && ( $prob_cutoff < 0 || $prob_cutoff > 1 ) ){

die "Error: P quality cutoff must be between 0 and 1";

}elsif( defined( $phrd_cutoff ) && $phrd_cutoff < 0 ){

die "Error: Phred quality cutoff must be greater than or equal to 0";

}

if( !`which R 2> err.log` ){

print STDERR "Warning: Subsidiary program R not found. Histogram will not be produced.\n";

}

`rm err.log`;

foreach my $input_file ( @files ){

open( INPUT, "<$input_file" ) or die "Error: Failure opening $input_file for reading: $!\n";

my @filepath = split( /\//, $input_file );

my $filename = $filepath[$#filepath];

if( !$user_defined ){

$format = "";

}

if( !$format ){

$format = &get_format(*INPUT, $automatic_detection_lines);

if( !$format ){

die "Error: File format cannot be determined\n";

}

}

my %dict_q_to_Q;

%dict_q_to_Q=&q_to_Q();

if( $format eq "sanger" ){

$poor_quality_char = "!";

}elsif( $format eq "solexa" ){

$poor_quality_char = ";";

}elsif( $format eq "illumina" ){

$poor_quality_char = "@";

}

if( $roche ){

print STDOUT "User defined format: Roche 454, Sanger FASTQ format\n";

}elsif( $format eq "sanger" ){

if( $user_defined ){

print STDOUT "User defined format: Sanger FASTQ format\n";

}else{

print STDOUT "Automatic format detection: Sanger FASTQ format\n";

}

}elsif( $format eq "solexa" ){

if( $user_defined ){

print STDOUT "User defined format: Solexa FASTQ format, Illumina pipeline 1.2 or less\n";

}else{

print STDOUT "Automatic format detection: Solexa FASTQ format, Illumina pipeline 1.2 or less\n";

}

}elsif( $format eq "illumina" ){

if( $user_defined ){

print STDOUT "User defined format: Illumina FASTQ format, Illumina pipeline 1.3+\n";

}else{

print STDOUT "Automatic format detection: Illumina FASTQ format, Illumina pipeline 1.3+\n";

}

}

if( defined( $phrd_cutoff ) ){

$ascii_cutoff = &Q_to_q( $phrd_cutoff );

$prob_cutoff = sprintf("%.5f", &Q_to_p( $phrd_cutoff ));

}else{

$ascii_cutoff = &Q_to_q( &p_to_Q( $prob_cutoff ) );

}

my $threshold = 0;

if( $bwa ){

if( defined( $phrd_cutoff ) ){

$threshold = $phrd_cutoff;

}else{

$threshold = &p_to_Q( $prob_cutoff );

}

}

my $output_file;

if ( $directory ){

# remove any trailing '/'

$directory =~ s/\/\z//;

my $file_name = $filename . ".trimmed";

$output_file = File::Spec->catpath( undef, $directory, $file_name );

}else{

$output_file = $filename . ".trimmed";

}

if( -e $output_file ){

die "Error: Output file $output_file already exists: $!\n";

}

open( OUTPUT, ">$output_file" )

or die "Error: Failure opening $output_file for writing: $!\n";

my @segment_hist;

my %hash=();

my $segment_sum = 0;

my $segment_count = 0;

my $original_length;

my $seq_count = 0;

while( <INPUT> ){

my $ID1 = $_;

if( substr( $ID1, 0 , 1) ne "@" ){

die "Error: Input file not in correct FASTQ format at seq ID $ID1\n";

}

chomp( my $seq_string = <INPUT> );

my $ID2 = <INPUT>;

if( substr( $ID2, 0 , 1) ne "+" ){

die "Error: Input file not in correct FASTQ format at qual ID $ID2\n";

}

chomp( my $quality_string = <INPUT> );

$original_length = length $seq_string;

my $cutoff_hit = 0;

my $best_start_index = 0;

my $best_length = 0;

my $current_start = 0;

my $bad_first = 0;

if( $bwa ){

my @qual = split(//, $quality_string );

for( my $i = 0; $i < scalar @qual; $i++ ){

$qual[$i] = $dict_q_to_Q{$qual[$i]};

}

if( !$qual[0] ){

$bad_first = 1;

$best_length = 0;

}elsif( $qual[0] < $threshold ){

$bad_first = 1;

$best_length = &bwa_trim( $threshold, \@qual );

}else{

$best_length = &bwa_trim( $threshold, \@qual );

}

}else{

for( my $i = 0; $i < $original_length; $i++ ){

if( substr($quality_string, $i, 1) le $ascii_cutoff ){

$cutoff_hit = 1;

my $current_segment_length = $i - $current_start;

if( $current_segment_length > $best_length ){

$best_length = $current_segment_length;

$best_start_index = $current_start;

}

$current_start = $i + 1;

}elsif( $i == $original_length - 1){

my $current_segment_length = ($i + 1) - $current_start;

if( $current_segment_length > $best_length ){

$best_length = $current_segment_length;

$best_start_index = $current_start;

}

}

}

if( !$cutoff_hit ){

$best_length = $original_length;

}

}

if( !defined($segment_hist[ $best_length ] ) ){

$segment_hist[ $best_length ] = 0;

}

$segment_hist[ $best_length ]++;

$segment_sum += $best_length;

$segment_count++;

if (exists $hash{$best_length}) {

$hash{$best_length}+=1;

}

else{

$hash{$best_length}=1

}

if( $bwa ){

if( $best_length <= 1 && $bad_first ) {

$seq_string = "N";

$quality_string = $poor_quality_char;

}else{

$seq_string = substr($seq_string, 0, $best_length);

$quality_string = substr($quality_string, 0, $best_length);

}

}else{

if ($best_length <= 0) {

$seq_string = "N";

$quality_string = $poor_quality_char;

} else {

$seq_string = substr($seq_string, $best_start_index, $best_length);

$quality_string = substr($quality_string, $best_start_index, $best_length);

}

}

print OUTPUT $ID1, $seq_string, "\n", $ID2, $quality_string, "\n";

}

my $segment_mean = sprintf( "%.1f", $segment_sum / $segment_count );

my $halfway_index = $segment_count / 2;

my $current_sum = 0;

my $current_index = 0;

my $median_index1;

my $median_index2;

while( !defined( $median_index1 ) || !defined( $median_index2 ) ){

if( defined( $segment_hist[ $current_index ] ) ){

$current_sum += $segment_hist[ $current_index ];

}

if( $current_sum > $halfway_index ){

if( !defined( $median_index1 ) ){

$median_index1 = $current_index;

}

if( !defined( $median_index2 ) ){

$median_index2 = $current_index;

}

}elsif( $current_sum == $halfway_index && !defined( $median_index1 ) ){

$median_index1 = $current_index;

}

$current_index++;

}

$current_index--;

my $segment_median;

if( $segment_count % 2 == 1){

$segment_median = $median_index1;

}else{

$segment_median = sprintf( "%.0f", ( ( $median_index1 + $median_index2 ) / 2 ) );

}

print STDOUT "Info: $output_file: mean segment length = $segment_mean, median segment length = $segment_median\n";

close INPUT or die "Error: Cannot close $input_file: $!";

close OUTPUT or die "Error: Cannot close $output_file; $!";

my $segments_filename;

if ( $directory ){

$segments_filename="$directory/$filename.trimmed_segments";

}

else{

$segments_filename="$filename.trimmed_segments";

}

open(SEGMENTS, ">$segments_filename");

print SEGMENTS "Read_length\tProportion_of_reads\n";

my $i;

for ($i=0;$i <= $original_length; $i++){

if (exists $hash{$i}){

my $percentage=$hash{$i}/$segment_count;

print SEGMENTS "$i\t$percentage\n"; }

else{ print SEGMENTS "$i\t0\n";

}

}

close SEGMENTS or die "Error: Cannot close $segments_filename; $!";

}
