# Supplementary material for: Protein Domain Analysis of Genomic Sequence Data Reveals Regulation of LRR Related Domains in Plant Transpiration in Ficus
Source: PLoS One. 2014 Sep 30;9(9):e108719. doi: 10.1371/journal.pone.0108719 (PMC4182558; doi:10.1371/journal.pone.0108719)
Supplement: Script S2 — Perl program used to delete the fastq reads which have length less than a specific value as well as to erase the “orphange” reads (single reads without pair). (DOCX) [file pone.0108719.s003.docx]

**Script S2**

::::::::::::::

fastqTrim_Length.pl: This program was used to delete the fastq reads which have length less than a specific value as well as to erase the “orphanage” reads (single reads without pair)

::::::::::::::

#!/usr/bin/env perl

use strict;

use warnings;

use Getopt::Long;

use File::Spec;

my $length = 25;

my $paired = 0;

my $directory;

my $usage = "

$0 one single-end or two paired-end FASTQ files [-l|length 25] [-d|directory path]\n

-l|length length cutoff [defaults to 25 nucleotides]

-d|directory path to directory where output files are saved

\n";

GetOptions(

"l|length=i" => \$length,

"d|directory=s" => \$directory

);

my @files = @ARGV;

if( !$files[0] || length(@files) > 2 ){

die $usage;

}

if( scalar(@files) == 2 ){

$paired = 1;

}

unless( -e $files[0] ){

die "error: file $files[0] does not exist\n";

}

open( FIRST, "<$files[0]" )

or die "error: failure opening $files[0] for reading: $!\n";

if( $paired ){

unless( -e $files[1] ){

die "error: file $files[1] does not exist\n";

}

open( SECOND, "<$files[1]" )

or die "error: failure opening $files[1] for reading: $!\n";

}

my $first_line;

my $second_line;

$first_line = <FIRST>;

if( substr($first_line, 0, 1) ne "@" ){

die "error: $files[0] does not appear to be in FASTQ format\n";

}

if( $paired ){

$second_line = <SECOND>;

if( substr($second_line, 0, 1) ne "@" ){

die "error: $files[1] does not appear to be in FASTQ format\n";

}

}

my $first_id;

my $second_id;

if( $paired ){

if( $first_line !~ /\S+\s\S+/ ){

if( $first_line =~ /(\S*)\/\S*/ ){

$first_id = $1;

}else{

$first_id = $first_line;

}

}elsif( $first_line =~ /\S+\s\S+/ ){

my @first_line_elements = split( /\s+/, $first_line );

pop @first_line_elements;

$first_id = join( " ", @first_line_elements );

}else{

$first_id = $first_line;

}

if( $second_line !~ /\S+\s\S+/ ){

if( $second_line =~ /(\S*)\/\S*/ ){

$second_id = $1;

}else{

$second_id = $second_line;

}

}elsif( $second_line =~ /\S+\s\S+/ ){

my @second_line_elements = split( /\s+/, $second_line );

pop @second_line_elements;

$second_id = join( " ", @second_line_elements );

}else{

$second_id = $second_line;

}

if( $first_id ne $second_id ){

die "error: files $files[0] and $files[1] do not seem to be paired\n";

}

}

if( $paired ){

my $first_line_counter = 0;

my $second_line_counter = 0;

$first_line_counter++ while <FIRST>;

$second_line_counter++ while <SECOND>;

if( $first_line_counter != $second_line_counter ){

die "error: files $files[0] and $files[1] appear to be different lengths\n";

}

}

seek(FIRST, 0, 0);

if( $paired ){

seek(SECOND, 0, 0);

}

my $single_file;

if ( $directory ){

$directory =~ s/\/\z//;

my @file_ending_elements = split(/\//, $files[0]);

my $item = scalar @file_ending_elements - 1;

my $file_name = $file_ending_elements[$item] . ".single";

$single_file = File::Spec->catpath( undef, $directory, $file_name );

}else{

$single_file = $files[0] . ".single";

}

if( -e $single_file ){

die "error: file $single_file already exists\n";

}

open( SINGLE, ">$single_file" )

or die "error: failure opening $single_file for writing: $!\n";

my $discard_file;

if ( $directory ){

$directory =~ s/\/\z//;

my @file_ending_elements = split(/\//, $files[0]);

my $item = scalar @file_ending_elements - 1;

my $file_name = $file_ending_elements[$item] . ".discard";

$discard_file = File::Spec->catpath( undef, $directory, $file_name );

}else{

$discard_file = $files[0] . ".discard";

}

if( -e $discard_file ){

die "error: file $discard_file already exists\n";

}

open( DISCARD, ">$discard_file" )

or die "error: failure opening $discard_file for writing: $!\n";

my $paired_file1;

my $paired_file2;

if( $paired ){

if ( $directory ){

$directory =~ s/\/\z//;

my @file_ending_elements = split(/\//, $files[0]);

my $item = scalar @file_ending_elements - 1;

my $file_name = $file_ending_elements[$item] . ".paired1";

$paired_file1 = File::Spec->catpath( undef, $directory, $file_name );

}else{

$paired_file1 = $files[0] . ".paired1";

}

if( -e $paired_file1 ){

die "error: file $paired_file1 already exists\n";

}

open( PAIRED1, ">$paired_file1" )

or die "error: failure opening $paired_file1 for writing: $!\n";

if ( $directory ){

$directory =~ s/\/\z//;

my @file_ending_elements = split(/\//, $files[0]);

my $item = scalar @file_ending_elements - 1;

my $file_name = $file_ending_elements[$item] . ".paired2";

$paired_file2 = File::Spec->catpath( undef, $directory, $file_name );

}else{

$paired_file2 = $files[0] . ".paired2";

}

if( -e $paired_file2 ){

die "error: file $paired_file2 already exists\n";

}

open( PAIRED2, ">$paired_file2" )

or die "error: failure opening $paired_file2 for writing: $!\n";

}

my $count_p1=0;

my $count_p2=0;

my $count_d=0;

my $count_s=0;

until( eof(FIRST) ){

chomp( my $first_header_line1 = <FIRST> );

chomp( my $first_sequence_line = <FIRST> );

chomp( my $first_header_line2 = <FIRST> );

chomp( my $first_quality_line = <FIRST> );

my $second_header_line1;

my $second_sequence_line;

my $second_header_line2;

my $second_quality_line;

if( $paired ){

chomp( $second_header_line1 = <SECOND> );

chomp( $second_sequence_line = <SECOND> );

chomp( $second_header_line2 = <SECOND> );

chomp( $second_quality_line = <SECOND> );

}

if( $paired ){

my $first_header_id;

my $second_header_id;

if( $first_header_line1 !~ /\S+\s\S+/ ){

if( $first_header_line1 =~ /(\S*)\/\S*/ ){

$first_header_id = $1;

}else{

$first_header_id = $first_header_line1;

}

}elsif( $first_header_line1 =~ /\S+\s\S+/ ){

my @first_header_line_elements = split( /\s+/, $first_header_line1 );

pop @first_header_line_elements;

$first_header_id = join( " ", @first_header_line_elements );

}else{

$first_header_id = $first_header_line1;

}

# second of pair

if( $second_header_line1 !~ /\S+\s\S+/ ){

if( $second_header_line1 =~ /(\S*)\/\S*/ ){

$second_header_id = $1;

}else{

$second_header_id = $second_header_line1;

}

}elsif( $second_header_line1 =~ /\S+\s\S+/ ){

my @second_header_line_elements = split( /\s+/, $second_header_line1 );

pop @second_header_line_elements;

$second_header_id = join( " ", @second_header_line_elements );

}else{

$second_header_id = $second_header_line1;

}

if( $first_header_id ne $second_header_id ){

die "error: header lines in $files[0] and $files[1] do not seem to be paired\n";

}

}

if( $paired ){

if( length($first_sequence_line) >= $length && length($second_sequence_line) >= $length ){

print PAIRED1 $first_header_line1, "\n", $first_sequence_line, "\n", $first_header_line2, "\n", $first_quality_line, "\n";

$count_p1+=1;

print PAIRED2 $second_header_line1, "\n", $second_sequence_line, "\n", $second_header_line2, "\n", $second_quality_line, "\n";

$count_p2+=1;

}

elsif( length($first_sequence_line) < $length && length($second_sequence_line) < $length ){

print DISCARD $first_header_line1, "\n", $first_sequence_line, "\n", $first_header_line2, "\n", $first_quality_line, "\n";

$count_d+=1;

print DISCARD $second_header_line1, "\n", $second_sequence_line, "\n", $second_header_line2, "\n", $second_quality_line, "\n";

$count_d+=1;

}

elsif( length($first_sequence_line) < $length && length($second_sequence_line) >= $length ){

print DISCARD $first_header_line1, "\n", $first_sequence_line, "\n", $first_header_line2, "\n", $first_quality_line, "\n";

$count_d+=1;

print SINGLE $second_header_line1, "\n", $second_sequence_line, "\n", $second_header_line2, "\n", $second_quality_line, "\n";

$count_s+=1;

}

elsif( length($first_sequence_line) >= $length && length($second_sequence_line) < $length ){

print SINGLE $first_header_line1, "\n", $first_sequence_line, "\n", $first_header_line2, "\n", $first_quality_line, "\n";

$count_s+=1;

print DISCARD $second_header_line1, "\n", $second_sequence_line, "\n", $second_header_line2, "\n", $second_quality_line, "\n";

$count_d+=1;

}

}

else{

if( length($first_sequence_line) >= $length ){

print SINGLE $first_header_line1, "\n", $first_sequence_line, "\n", $first_header_line2, "\n", $first_quality_line, "\n";

$count_s+=1;

}else{

print DISCARD $first_header_line1, "\n", $first_sequence_line, "\n", $first_header_line2, "\n", $first_quality_line, "\n";

$count_d+=1;

}

}

}

my @name = split(/\./, $single_file);

my $summaryname= join '.', @name[0..$#name-1];

my $outputname = $summaryname.".summary.txt";

open(FILEOUT, ">$outputname");

if( $paired ){

print FILEOUT "paired1\t$count_p1\n", "paired2\t$count_p2\n", "single\t$count_s\n", "discard\t$count_d\n";}

else{

print FILEOUT "single\t$count_s\n", "discard\t$count_d\n";}

close FILEOUT;

close FIRST or die "error: failure closing $files[0]: $!\n";

if( $paired ){

close SECOND or die "error: failure closing $files[1]: $!\n";

}

close SINGLE or die "error: failure closing $single_file: $!\n";

close DISCARD or die "error: failure closing $discard_file: $!\n";

if( $paired ){

close PAIRED1 or die "error: failure closing $paired_file1: $!\n";

close PAIRED2 or die "error: failure closing $paired_file2: $!\n";

}

exit 0 or die "error: $0 ended abnormally: $!\n";
