# Supplementary material for: Protein Domain Analysis of Genomic Sequence Data Reveals Regulation of LRR Related Domains in Plant Transpiration in Ficus
Source: PLoS One. 2014 Sep 30;9(9):e108719. doi: 10.1371/journal.pone.0108719 (PMC4182558; doi:10.1371/journal.pone.0108719)
Supplement: Script S3 — Perl programs used for dealing with the assembly files which were created by Phrap as well as for making statistic analysis. (DOCX) [file pone.0108719.s004.docx]

**Script S3**

These are programs used for dealing with the assembly files which were created by Phrap as well as for making statistical analysis

::::::::::::::

delete_short_aln_multinput.pl

::::::::::::::

#!/usr/bin/env perl

if (-d "aln_keep"){die "already exist dir aln_keep\n";}

system ("mkdir aln_keep");

open OUT, ">delete.txt";

use Bio::SeqIO;

@ids=qx{ls};

foreach $input(@ids){

chomp $input;

next unless $input =~ /\.aln/;

$in = Bio::SeqIO -> new (-file => "$input", -format => 'Fasta');

$seqobj = $in -> next_seq();

$seq = $seqobj -> seq;

$seq=~s/n//ig;

$seg=~s/-//g;

if(length($seq)>=250){system("cp $input aln_keep/");}

else{print OUT "rm -f $input\necho Delete $input.\n"; }

}

::::::::::::::

length.pl

::::::::::::::

#!/usr/bin/env perl

use Bio::SeqIO;

$in = Bio::SeqIO -> newFh (-fh => \*ARGV, -format => 'Fasta');

while (<$in>){

$id=$_->id;

$seq=$_->seq;

print ">",$id," ",length($seq),"\n";

}

::::::::::::::

N.pl

::::::::::::::

#!/usr/bin/perl

$p=shift;

$source=shift;

use Bio::SeqIO;

$in = Bio::SeqIO -> new(-file=>$source, -format=>'Fasta');

@seq=();

$total=0;

while(my $stream = $in->next_seq()){

$sequence=$stream->seq;

$len=length($sequence);

push (@seq,$len);

$total=$total+$len;

@seq = sort {$a <=> $b} @seq;

$N=0;

for ($i=0;$i<scalar@seq;$i++){

$N=$N+$seq[$i];

if ($N>=$total*(100-$p)/100){print "N$p:$seq[$i]\n"; exit;}

}

}

::::::::::::::

phrap_all_lang_parameters.pl

::::::::::::::

#!/usr/bin/env perl

$input=shift;

$default_qual=15; #1

$trim_start=0; #2

$force_level=0; #3

$bypass_level=1; #4

$maxgap=30; #5

$repeat_stringency=0.7; #6

$nodeseg=8; #7

$nodespace=4; #8

$qual_show=20; #9

$max_subclone_size=5000; #10

$trim_score=20; #11

$trim_penalty=-2; #12

$trim_qual=13; #13

$confirm_length=8; #14

$confirm_trim=1; #15

$confirm_penalty=-5; #16

$confirm_score=30; #17

$indexwordsize=10; #18

open IN,"<$input";

while (<IN>){

chomp;

system ("phrap $_ -ace -maxgap $maxgap -repeat_stringency $repeat_stringency -node_space $nodespace -node_seg $nodeseg -tags -default_qual $default_qual -qual_show $qual_show -retain_duplicates -max_subclone_size $max_subclone_size -trim_penalty $trim_penalty -trim_score $trim_score -trim_qual $trim_qual -confirm_length $confirm_length -confirm_trim $confirm_trim -confirm_penalty $confirm_penalty -confirm_score $confirm_score -indexwordsize $indexwordsize -trim_start $trim_start -forcelevel $force_level -bypasslevel $bypass_level");

`rm -f *.contigs*`; `rm -f *.log`; `rm -f *.problems*`; `rm -f *.singlets`;

}

::::::::::::::

pickgroup_useModule.pl

::::::::::::::

#!/usr/bin/perl

# pick out a list of genes.

$input=$ARGV[0];

$source=$ARGV[1];

use Bio::SeqIO;

open(IN,"<$input");

while($line=<IN>){

chomp $line;

$line=~s/\s.*//;

$line=~s/>//;

$in = new Bio::SeqIO(-file => $source, -format => 'Fasta');

while($stream=$in->next_seq()){

if($line eq $stream->id){

print ">",$stream->id," ",$stream->desc,"\n",$stream->seq,"\n";

last;

}

}

}

close IN;

::::::::::::::

pick_notin.pl

::::::::::::::

#!/usr/sbin/perl

my $source = $ARGV[0];

my $known = $ARGV[1];

open (IN1, "<$source") || die "cannnot open \"$source\": $!";

while (my $line1 = <IN1>){

open (IN2, "<$known") || die "cannnot open \"$known\": $!";

chomp $line1;

$line1backup = $line1;

$line1 =~ s/\s//g;

my $match = 0;

while (my $line2 = <IN2>){

chomp $line2;

$line2 =~ s/\s//g;

if ($line1 eq $line2){$match = 1;}

if ($match==0){print "$line1backup\n";}

}

close IN2;

}

close IN1;

::::::::::::::

pick.pl

::::::::::::::

#!/usr/sbin/perl

my $input = $ARGV[0];

my $source = $ARGV[1];

open (IN, "<$source") || die "cannnot open \"$source\": $!";

my $match;

chomp($input);

while (my $line = <IN>){

chomp $line;

if ($line =~ />/){$match = 0;}

if ($line =~ /$input/){$match = 1;}

if ($match==1){print "$line\n";}

}

close IN;

::::::::::::::

split_fasta_each.pl

::::::::::::::

#!/usr/bin/env perl

use Bio::SeqIO;

$in = Bio::SeqIO -> newFh (-fh => \*ARGV, -format => 'Fasta');

while (<$in>){

$id = $_ -> id;

$desc = $_ -> desc;

$seq = $_ -> seq;

$output=$id.".fa";

open OUT, ">$output";

print OUT ">",$id," ",$desc,"\n",$seq,"\n";

}

::::::::::::::

write_acealn-nonqual_multinput.pl

::::::::::::::

#!/usr/bin/env perl

if (-d "aln"){die "already exist dir aln\n";}

system("mkdir aln");

use Bio::Assembly::IO;

@ids=qx{ls};

foreach $input(@ids){

chomp $input;

next unless $input =~ /polybayes\.ace/;

$input2=$input;

$input2=~s/\..*//g;

print $input2,"\n";

$assembly = Bio::Assembly::IO -> new (-file =>$input, -format =>'ace') -> next_assembly;

foreach $contig ($assembly->all_contigs){

$contig_id=$contig->id;

$goal="$input2"."_contig"."$contig_id".".aln";

$consensus=$contig->get_consensus_sequence;

$consensus_len=$contig->get_consensus_length;

open OUT, ">$goal";

print OUT ">Contig",$contig_id,"|Length:",$consensus_len,"|No_of_Seq:",$contig->no_sequences,"\n",$consensus->seq,"\n";

foreach $seq ($contig->each_seq){

$seqid=$seq->id;

$seqlen=$seq->length;

$seqfeat=$contig->get_seq_coord($seq);

$seqstart=$seqfeat->start;

$seqstrand=$seqfeat->strand;

$alignseq=$seq->seq;

print OUT ">",$seqid,"|strand:",$seqstrand,"|start:",$seqstart,"\n";

if ($seqstart<=0){

$trim=1-$seqstart;

$alignseq = substr ($alignseq,$trim,length($alignseq)-$trim);

}

for ($i=1;$i<$seqstart;$i++){$alignseq="?".$alignseq;}

for ($i=1;$i<$consensus_len-$seqstart-$seqlen+2;$i++){$alignseq=$alignseq."?";}

$alignseq=substr($alignseq,0,$consensus_len);

print OUT $alignseq,"\n";

}

system ("mv $goal aln/");

}

}

::::::::::::::

write_acealn-nonqual_oneinput.pl

::::::::::::::

#!/usr/bin/env perl

$input=shift;

use Bio::Assembly::IO;

$assembly = Bio::Assembly::IO -> new (-file =>$input, -format =>'ace') -> next_assembly;

foreach $contig ($assembly->all_contigs){

$contig_id=$contig->id;

$consensus=$contig->get_consensus_sequence;

$consensus_len=$contig->get_consensus_length;

print ">Contig",$contig_id,"|Length:",$consensus_len,"|No_of_Seq:",$contig->no_sequences,"\n",$consensus->seq,"\n";

foreach $seq ($contig->each_seq){

$seqid=$seq->id;

$seqlen=$seq->length;

$seqfeat=$contig->get_seq_coord($seq);

$seqstart=$seqfeat->start;

$seqstrand=$seqfeat->strand;

$alignseq=$seq->seq;

print ">",$seqid,"|strand:",$seqstrand,"|start:",$seqstart,"\n";

if ($seqstart<=0){

$trim=1-$seqstart;

$alignseq = substr ($alignseq,$trim,length($alignseq)-$trim);

}

for ($i=1;$i<$seqstart;$i++){$alignseq="?".$alignseq;}

for ($i=1;$i<$consensus_len-$seqstart-$seqlen+2;$i++){$alignseq=$alignseq."?";}

$alignseq=substr($alignseq,0,$consensus_len);

print $alignseq,"\n";

}

}
